# Supplementary material for: Learning algorithms allow for improved reliability and accuracy of global mean surface temperature projections
Source: Nat Commun. 2020 Jan 23;11:451. doi: 10.1038/s41467-020-14342-9 (PMC6978329; doi:10.1038/s41467-020-14342-9)
Supplement: Supplementary file 1 — Supplementary Information [file 41467_2020_14342_MOESM1_ESM.pdf]

Supplementary Information

Learning algorithms allows for improved reliability and accuracy of global mean surface temperature projections

Strobach et al.

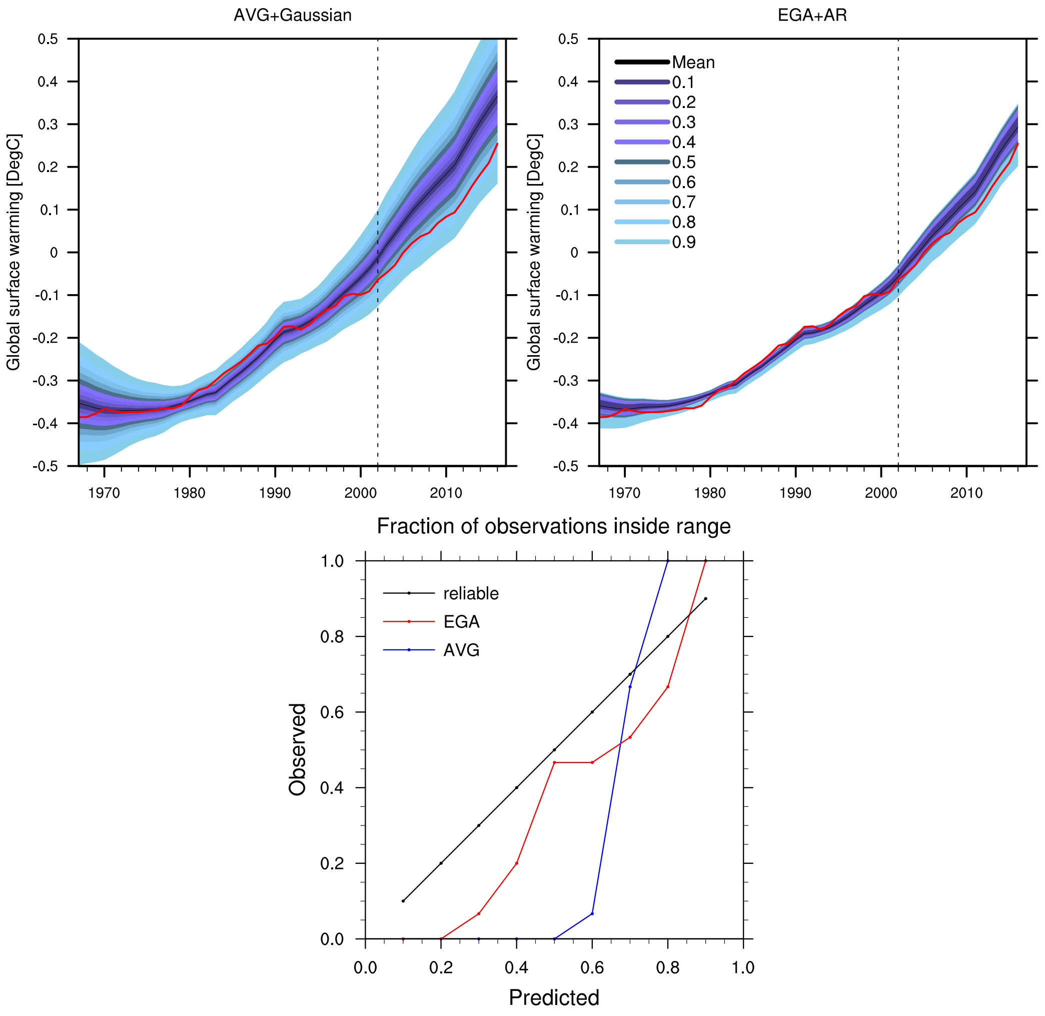

Supplementary Figure 1: **Top panels:** Global mean surface temperature change relative to 1986–2005 average. The black lines represent the ensemble mean, and the shadings represent different significance levels (0.1–0.9). The left part of each panel (to the left of the dashed vertical line) represents the learning period, and the right part of each panel represents the validation period. **Bottom panel:** Prediction period reliability diagram (the fraction of points within the estimated range vs. the expected fraction).

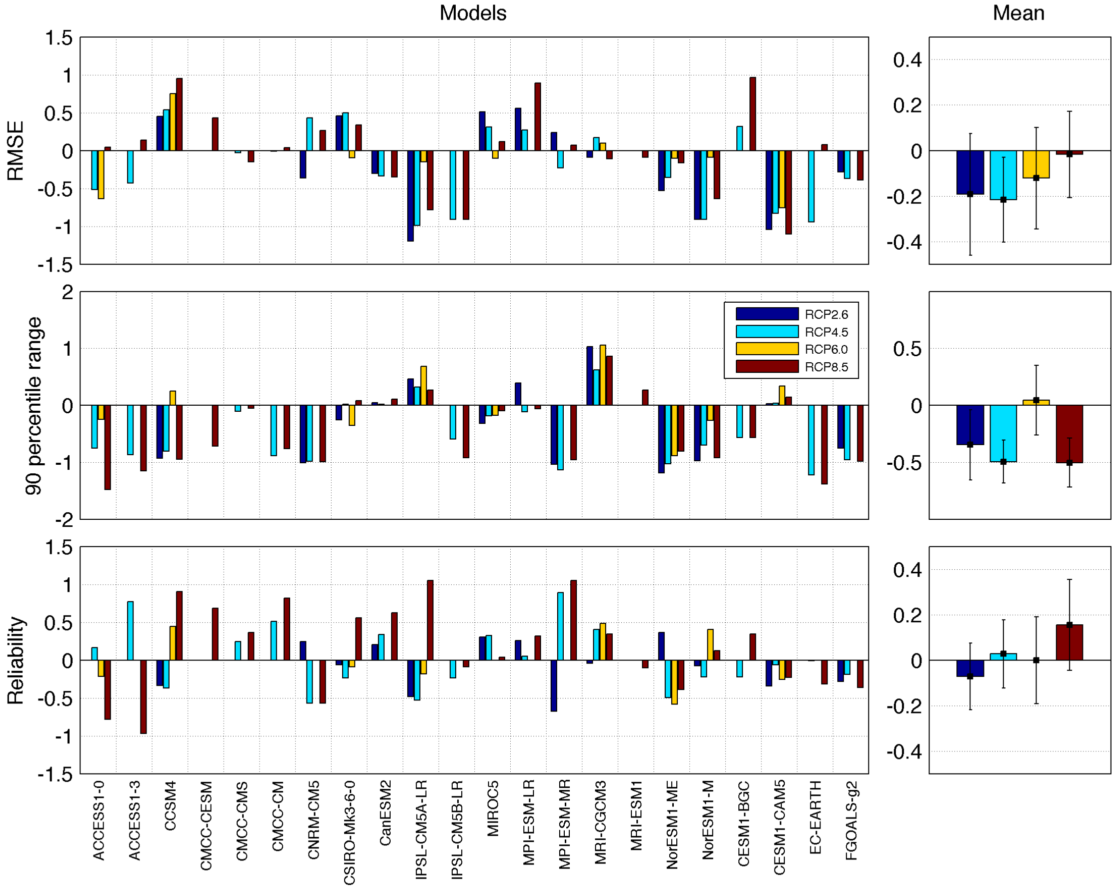

Supplementary Figure 2: **Left panels:** Relative RMSE ($\frac{RMSE_{EGA+AR}-RMSE_{AVG}}{(RMSE_{EGA+AR}+RMSE_{AVG})/2}$), relative 90 percentile range ($\frac{RAN_{EGA+AR}-RAN_{AVG}}{(RAN_{EGA+AR}+RAN_{AVG})/2}$), and relative reliability ($\frac{REL_{AVG}-REL_{EGA+AR}}{(REL_{EGA+AR}+REL_{AVG})/2}$) for the 22 models (the results for each model correspond to the out-of-sample test in which this model was used as observation) and 4 RCPs. **Right panels:** A summary of the results presenting the mean and the 90 percentile confidence interval (error bars).

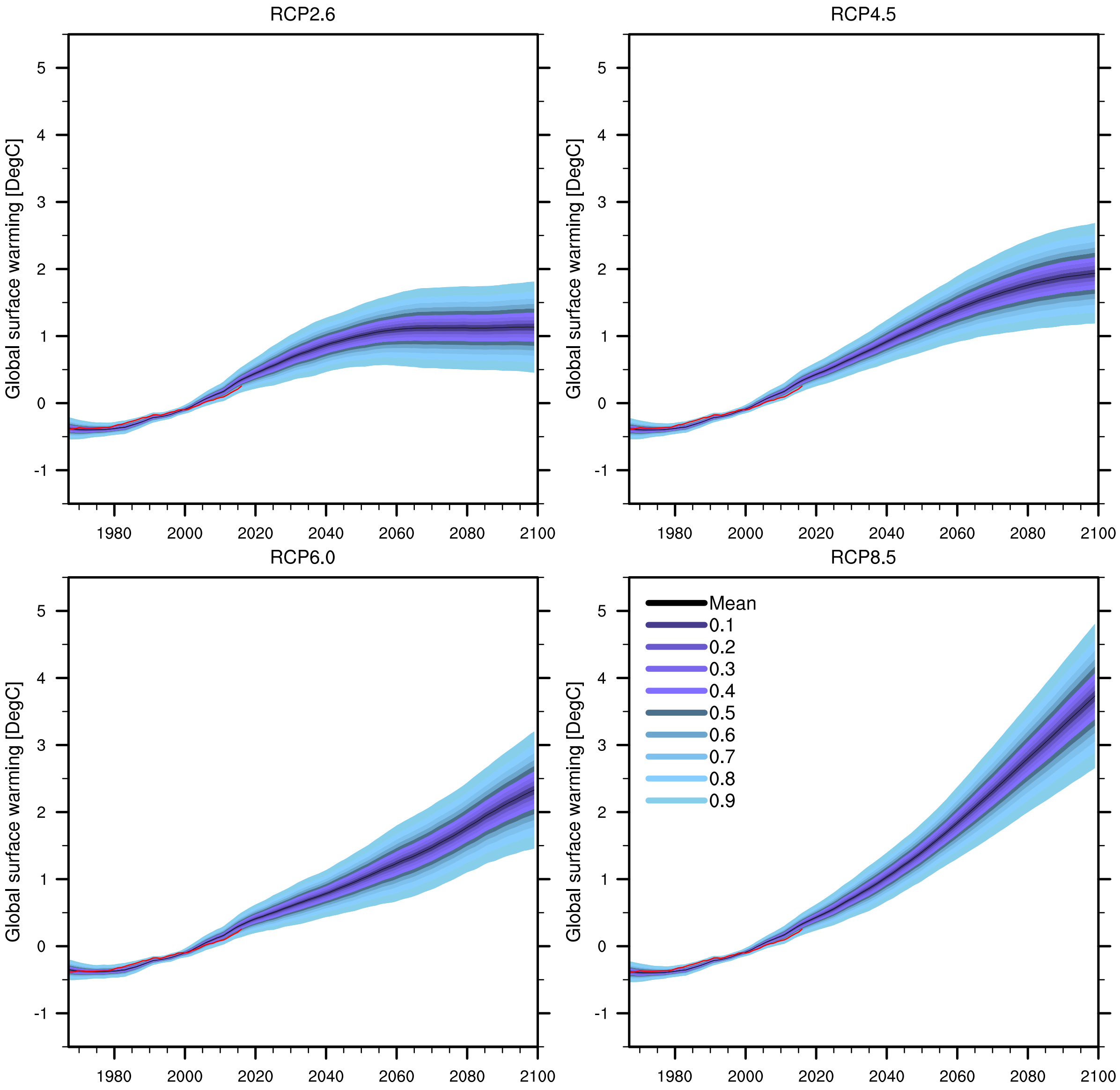

Supplementary Figure 3: Global mean surface temperature (GMST) change relative to the 1986–2005 average for the RCP scenarios included in CMIP5. The black lines represent the ensemble mean for the 20-year average GMST, and the shadings represent the uncertainty range, for different significance levels, based on the Gaussian assumption and equally weighted ensemble.

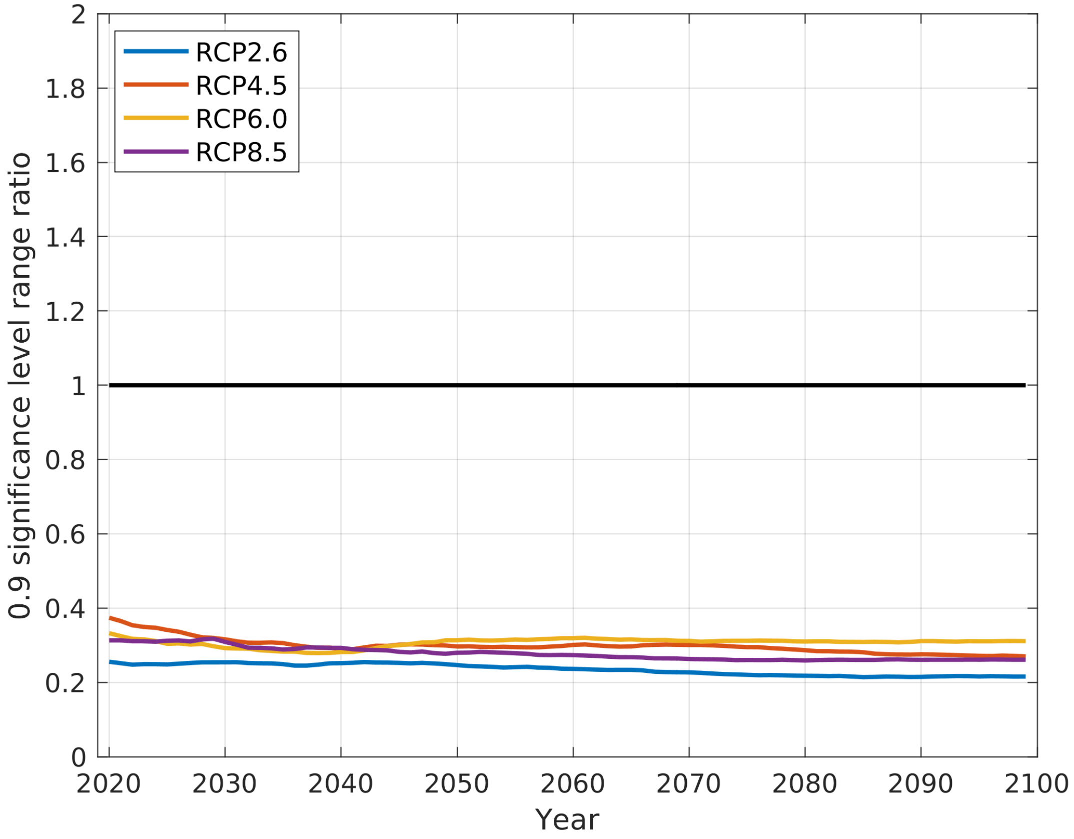

Supplementary Figure 4: The temporal variation of the ratio between the uncertainty range based on the EGA weighted ensemble and the AR method and the range based on the equally weighted ensemble and the Gaussian assumption for the 0.9 significance level. The different lines correspond to the different RCPs.

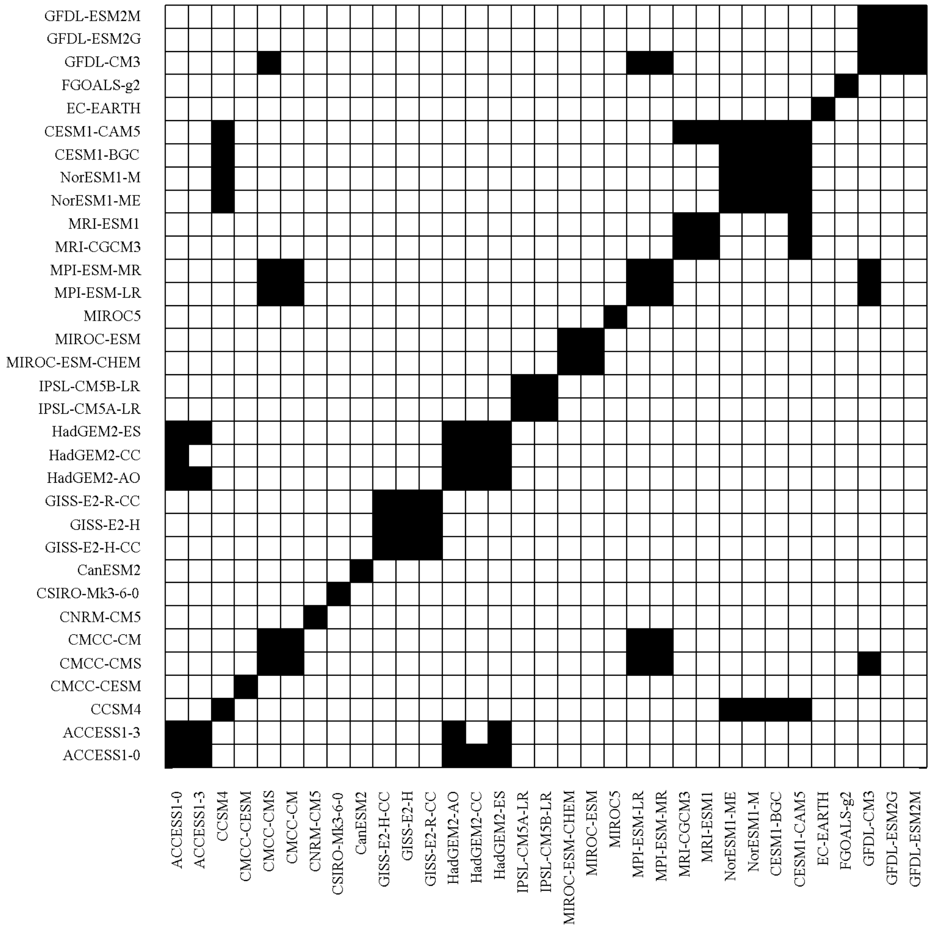

Supplementary Figure 5: Graphical representation of the excluded models in the out-of-sample test. Black squares in each column (row) represent models excluded from the projecting ensemble in the out-of-sample test when the model in that column (row) was used as observations.

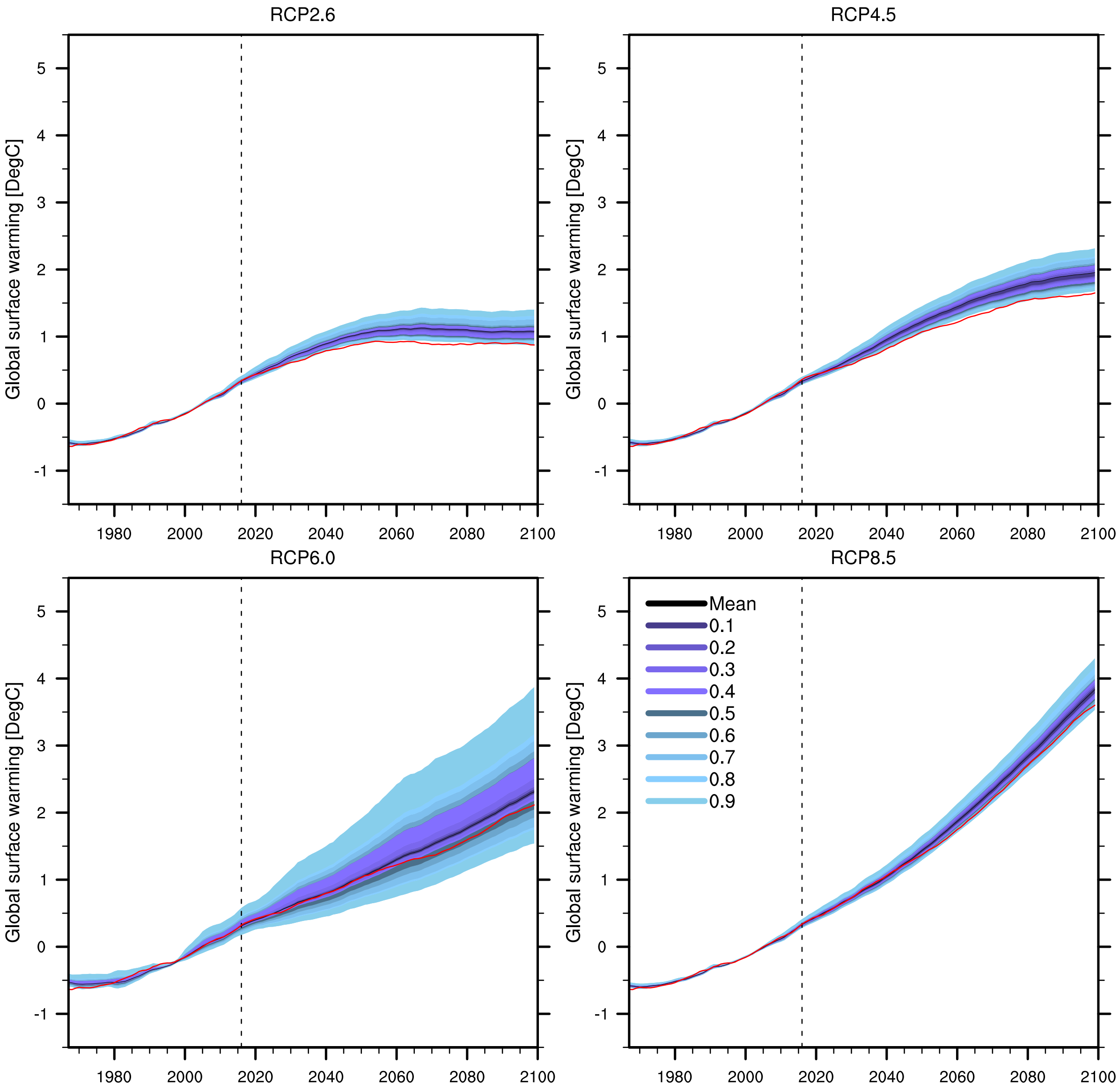

Supplementary Figure 6: **Top panels:** Global mean surface temperature change relative to 1986–2005 average. The black lines represent the ensemble mean, and the shadings represent different significance levels (0.1–0.9). The left part of each panel (to the left of the dashed vertical line) represents the learning period, and the right part of each panel represents the validation period. Red line represents the CCSM4 model, which functions as the out-of-sample observations.

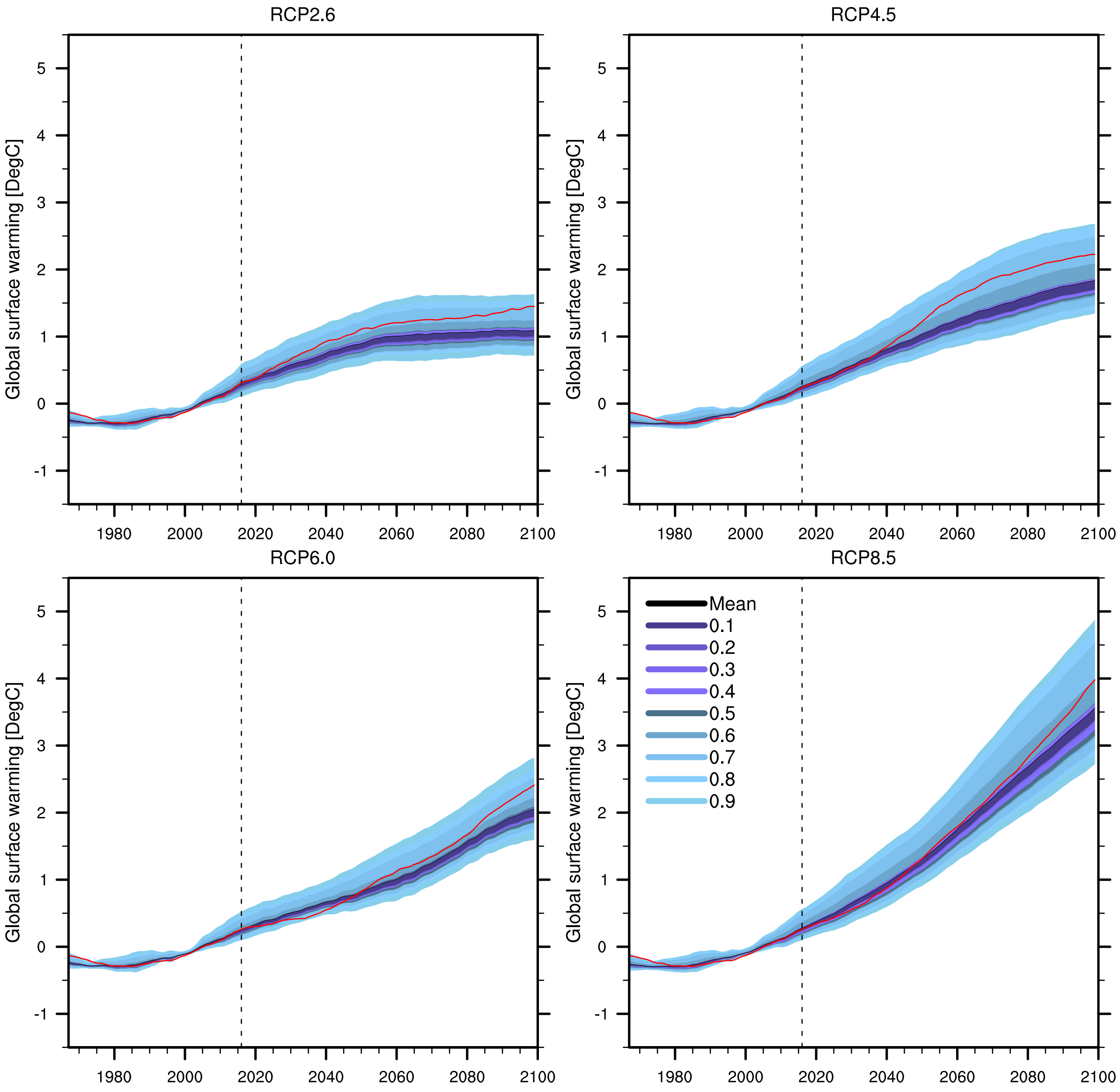

Supplementary Figure 7: **Top panels:** Global mean surface temperature change relative to 1986–2005 average. The black lines represent the ensemble mean, and the shadings represent different significance levels (0.1–0.9). The left part of each panel (to the left of the dashed vertical line) represents the learning period, and the right part of each panel represents the validation period. Red line represents the CSIRO-Mk3-6-0 model, which functions as the out-of-sample observations.

Supplementary Table 1: The ratio between the projected GMST uncertainty ranges based on the EGA and AR and the equally weighted and Gaussian estimations. The values represent the ratio between the ranges for two different periods of 20 years as denoted. The different columns correspond to different RCPs, and the different rows correspond to different confidence levels.

	2046–2065				2080–2099			
c	RCP2.6	RCM4.5	RCP6.0	RCP8.5	RCP2.6	RCM4.5	RCP6.0	RCP8.5
0.9	0.23	0.3	0.32	0.27	0.22	0.27	0.31	0.26
0.8	0.18	0.25	0.25	0.22	0.17	0.23	0.24	0.22
0.7	0.19	0.27	0.26	0.26	0.17	0.24	0.26	0.26
0.6	0.16	0.23	0.21	0.2	0.15	0.21	0.21	0.2
0.5	0.17	0.21	0.24	0.17	0.16	0.19	0.24	0.17
0.4	0.18	0.24	0.27	0.21	0.17	0.22	0.27	0.2
0.3	0.18	0.27	0.3	0.25	0.16	0.24	0.29	0.24
0.2	0.18	0.3	0.22	0.23	0.16	0.27	0.21	0.23
0.1	0.24	0.35	0.2	0.33	0.22	0.32	0.19	0.33

Supplementary Table 2: The uncertainty range of the projected GMST based on the EGA weighting and the AR estimation. The values correspond to two different periods of 20 years. The different columns correspond to different RCPs, and the different rows correspond to different confidence levels.

	2046–2065				2080–2099			
c	RCP2.6	RCM4.5	RCP6.0	RCP8.5	RCP2.6	RCM4.5	RCP6.0	RCP8.5
0.9	0.27	0.32	0.38	0.32	0.29	0.4	0.54	0.56
0.8	0.16	0.21	0.23	0.21	0.18	0.26	0.33	0.36
0.7	0.14	0.18	0.2	0.2	0.15	0.23	0.28	0.35
0.6	0.1	0.13	0.13	0.12	0.1	0.16	0.19	0.22
0.5	0.08	0.09	0.12	0.08	0.09	0.11	0.17	0.15
0.4	0.07	0.08	0.1	0.08	0.07	0.1	0.15	0.14
0.3	0.05	0.07	0.08	0.07	0.05	0.09	0.12	0.12
0.2	0.03	0.05	0.04	0.04	0.03	0.06	0.06	0.07
0.1	0.02	0.03	0.02	0.03	0.02	0.04	0.03	0.05

Supplementary Table 3: The uncertainty range of the projected GMST based on the equally weighted ensemble and the Gaussian estimation. The values correspond to two different periods of 20 years. The different columns correspond to different RCPs, and the different rows correspond to different confidence levels.

	2046–2065				2080–2099			
c	RCP2.6	RCM4.5	RCP6.0	RCP8.5	RCP2.6	RCM4.5	RCP6.0	RCP8.5
0.9	1.15	1.07	1.2	1.2	1.36	1.5	1.75	2.15
0.8	0.9	0.83	0.93	0.94	1.06	1.17	1.36	1.68
0.7	0.73	0.67	0.75	0.76	0.85	0.94	1.1	1.36
0.6	0.59	0.55	0.61	0.62	0.69	0.77	0.89	1.1
0.5	0.47	0.44	0.49	0.49	0.56	0.61	0.72	0.88
0.4	0.37	0.34	0.38	0.38	0.43	0.48	0.56	0.69
0.3	0.27	0.25	0.28	0.28	0.32	0.35	0.41	0.5
0.2	0.18	0.16	0.18	0.19	0.21	0.23	0.27	0.33
0.1	0.09	0.08	0.09	0.09	0.1	0.11	0.13	0.16

Supplementary Table 4: γ_u and γ_d for different RCPs and significance levels.

	RCP2.6		RCP4.5		RCP6.0		RCP8.5	
c	γ_u	γ_d	γ_u	γ_d	γ_u	γ_d	γ_u	γ_d
0.9	0.591	0.227	0.701	0.307	0.76	0.29	0.653	0.293
0.8	0.318	0.181	0.407	0.25	0.45	0.19	0.395	0.215
0.7	0.264	0.152	0.343	0.232	0.37	0.18	0.38	0.202
0.6	0.157	0.136	0.203	0.195	0.21	0.15	0.192	0.174
0.5	0.136	0.109	0.168	0.118	0.19	0.14	0.143	0.103
0.4	0.107	0.094	0.15	0.108	0.16	0.13	0.131	0.1
0.3	0.084	0.062	0.12	0.093	0.14	0.09	0.122	0.082
0.2	0.064	0.032	0.107	0.048	0.06	0.05	0.072	0.054
0.1	0.042	0.023	0.09	0.001	0.03	0.02	0.051	0.039

Supplementary Table 5: Skewness and kurtosis of the estimated (based on the EGA weighted ensemble and the AR estimation of the uncertainty) probability distribution of the 20-year average projected GMST. The cumulants are the same for all the years in the prediction period because the different years only differ in their average (which does not affect the central moments) and STD (which only sets the scale and does not affect the ratio between the moments of the probability distribution). The values of the correction factors $\gamma_{u,d}(c)$ are constant during the prediction period.

	skewness	excess kurtosis
RCP26	1.22	1.48
RCP45	0.95	0.73
RCP60	1.41	1.78
RCP85	1.03	0.8

Supplementary Table 6: Model Availability. Not all the models included in the CMIP5 projection data spanned the period of 1948–2100 considered in our study, and most of the models did not provide projections for all the RCPs. The table lists the models included in our ensemble for each RCP.

Model Name	RCP2.6	RCP4.5	RCP6.0	RCP8.5
ACCESS1-0	X	√	X	√
ACCESS1-3	X	√	X	√
CCSM4	√	√	√	√
CMCC-CESM	X	X	X	√
CMCC-CMS	X	√	X	√
CMCC-CM	X	√	X	√
CNRM-CM5	√	√	X	√
CSIRO-Mk3-6-0	√	√	√	√
CanESM2	√	√	X	√
GISS-E2-H-CC	X	√	X	√
GISS-E2-H	√	√	√	√
GISS-E2-R-CC	X	√	X	√
HadGEM2-AO	√	√	√	√
HadGEM2-CC	X	√	X	√
HadGEM2-ES	√	√	X	√
IPSL-CM5A-LR	√	√	√	√
IPSL-CM5B-LR	X	√	X	√
MIROC-ESM-CHEM	√	√	√	√
MIROC-ESM	√	√	√	√
MIROC5	√	√	√	√
MPI-ESM-LR	√	√	X	√
MPI-ESM-MR	√	√	X	√
MRI-CGCM3	√	√	√	√
MRI-ESM1	X	X	X	√
NorESM1-ME	√	√	√	√
NorESM1-M	√	√	√	√
CESM1-BGC	X	√	X	√
CESM1-CAM5	√	√	√	√
EC-EARTH	X	√	X	√
FGOALS-g2	√	√	X	√
GFDL-CM3	√	√	√	√
GFDL-ESM2G	√	√	√	√
GFDL-ESM2M	√	√	√	√
Total	21	31	15	33

Supplementary Table 7: The EGA weight assigned to each model and for each ensemble (different ensembles for different RCPs). These weights, assigned at the end of the learning period, remain time-independent during the projection period.

Model Name	RCP2.6	RCP4.5	RCP6.0	RCP8.5
ACCESS1	X	0.02	X	0.02
ACCESS1-3	X	0.04	X	0.04
CCSM4	0.02	0.01	0.07	0.01
CMCC-CESM	X	X	X	0.03
CMCC-CMS	X	0.01	X	0.01
CMCC-CM	X	0.02	X	0.02
CNRM-CM5	0.03	0.02	X	0.02
CSIRO-Mk3-6	0.04	0.05	0.03	0.03
CanESM2	0.01	X	X	X
GISS-E2-CC-H	X	0.02	X	0.02
GISS-E2-H	0.06	0.04	0.08	0.04
GISS-E2-CC-R	X	0.03	X	0.03
GISS-E2-R	X	X	X	X
HadGEM2-AO	0.01	0.01	0.02	0.01
HadGEM2-CC	X	0.02	X	0.02
HadGEM2-ES	0.02	0.01	X	0.01
IPSL-CM5A-LR	0.01	X	0.02	X
IPSL-CM5B-LR	X	0.06	X	0.05
MIROC-ESM-CHEM	0.07	0.05	0.11	0.05
MIROC-ESM	0.03	0.02	0.05	0.02
MIROC5	0.02	0.01	0.02	0.01
MPI-LR-ESM	0.06	0.03	X	0.03
MPI-ESM-MR	0.02	0.01	X	0.01
MRI-CGCM3	0.25	0.25	0.23	0.21
MRI-ESM1	X	X	X	0.08
NorESM1-ME	0.06	0.04	0.08	0.04
NorESM1-M	0.04	0.03	0.08	0.03
CESM1-BGC	X	0.01	X	0.01
CESM1-CAM5	0.09	0.07	0.12	0.06
EC-EARTH	X	0.02	X	0.01
FGOALS-g2	0.08	0.04	X	0.04
GFDL-CM3	0.01	0.01	0.01	0.01
GFDL-ESM2G	0.01	0.01	0.03	0.01
GFDL-ESM2M	0.03	0.02	0.05	0.02
Total	1	1	1	1

Supplementary Table 8: Quantiles for the 20-year average projected GMST. Values in parentheses were taken from the last IPCC report[1]. The quantiles are based on the EGA weighted ensemble and the AR estimation of the uncertainties. For all the RCPs and for both periods, we estimate a narrower distribution than the corresponding IPCC estimation.

	2046–2065			2080–2099 (2081–2100)		
	5%	50%	95%	5%	50%	95%
RCP2.6	0.92 (0.4)	0.99 (1.0)	1.19 (1.6)	0.96 (0.3)	1.04 (1.0)	1.26 (1.7)
RCP4.5	1.28 (0.9)	1.37 (1.4)	1.6 (2.0)	1.71 (1.1)	1.82 (1.8)	2.11 (2.6)
RCP6.0	1.16 (0.8)	1.26 (1.3)	1.54 (1.8)	2.08 (1.4)	2.23 (2.2)	2.63 (3.1)
RCP8.5	1.86 (1.4)	1.95 (2.0)	2.18 (2.6)	3.37 (2.6)	3.53 (3.7)	3.92 (4.8)

Supplementary Table 9: Ranges from the 5%–95% quantiles for the 20-year average projected GMST. The range estimated using the EGA weighted ensemble and the AR method for estimating the uncertainties is considerably smaller than the IPCC reported range for all the RCPs.

	5%–95%	
	2046–2065	2080–2099 (2081–2100)
RCP2.6	0.27 (1.2)	0.30 (1.4)
RCP4.5	0.32 (1.1)	0.40 (1.5)
RCP6.0	0.38 (1.0)	0.55 (1.7)
RCP8.5	0.32 (1.2)	0.55 (2.2)

Supplementary References

1. IPCC. *Climate Change 2013: The Physical Science Basis. Contribution of Working Group I to the Fifth Assessment Report of the Intergovernmental Panel on Climate Change* (Cambridge University Press, Cambridge, United Kingdom and New York, NY, USA, 2013).
